# The remarkable plethora of infestation-responsive Q-type C2H2 transcription factors in potato

**DOI:** 10.1186/s13104-018-3503-6

**Published:** 2018-06-19

**Authors:** Susan D. Lawrence, Nicole G. Novak

**Affiliations:** Invasive Insect Biocontrol and Behavior Lab, USDA-ARS, 10300 Baltimore Ave., BARC-West Bldg 007, Rm 301, Beltsville, MD 20705 USA

**Keywords:** Q-type C2H2 zinc finger proteins, *Solanum tuberosum*, Potato, *Manduca sexta* infestation

## Abstract

**Objective:**

Q-type C2H2 transcription factors (TF) play crucial roles in the plant response to stress, often leading to regulation of downstream genes required for tolerance to these challenges. An infestation-responsive Q-type C2H2 TF (StZFP2) is induced by wounding and infestation in potato. While mining the *Solanum tuberosum* Group Phureja genome for additional members of this family of proteins, five StZFP2-like genes were found on a portion of chromosome 11. The objective of this work was to differentiate these genes in tissue specificity and expression upon infestation.

**Results:**

Examination of different tissues showed that young roots had the highest amounts of transcripts for five of the genes. Expression of their transcripts upon excision or infestation by *Manduca sexta*, showed that all six genes were induced. Overall, each gene showed variations in its response to infestation and specificity for tissue expression. The six genes encode very similar proteins but most likely play unique roles in the plant response to infestation. In contrast, only two homologs have been identified in Arabidopsis and tomato. Overexpression of similar genes has led to enhanced tolerance to, for example, salinity, drought and pathogen stress. Discovery of these new StZFP2 homologs could provide additional resources for potato breeders.

**Electronic supplementary material:**

The online version of this article (10.1186/s13104-018-3503-6) contains supplementary material, which is available to authorized users.

## Introduction

Plants have an array of responses to environmental stresses such as excessive salinity, drought, pathogens and insects. Stressors are rapidly sensed by the plant, triggering hormonally mediated reactions [1]. Generally, attack by chewing insects lead to the induction of the jasmonic acid (JA) pathway and an array of direct and indirect defense compounds that presumably aid in protecting the plant under attack. Identification of an infestation responsive Q-type C2H2 transcription factors (TF), in potato, StZFP2 [2] led to our interest in this family of TFs. Overexpression of these TFs can result in increased tolerance to stress [3–6]. C2H2 zinc finger proteins (ZFP) contain two cysteine and two histidine residues that surround a zinc ion. While Englbrecht et al. described a total of 176 C2H2 ZFPs in Arabidopsis, 64 are found in the C1 family containing either one zinc finger domain (ZFD) or two to five dispersed ZFDs [7, 8]. The C1 group mostly contains the QALGGH motif, unique to plants and a crucial part of the 30 amino acid ZFD. Many of these proteins are induced by stress [8, 9]. Q-type is short hand for the QALGGH motif. Arabidopsis has 18 C1-2i proteins that contain two Q-type ZFDs, with the ZFDs acting as DNA-binding domains [8, 9]. Another important amino acid motif is the EAR domain, which is as an active repressor with either a DLN or LxLxL profile. The EAR domain was named from its initial discovery in the ethylene-responsive element binding factor or ERF family proteins. EAR stands for ERF-associated amphiphilic repression motif [10, 11]. The Q-type C2H2 ZFPs also contain two additional motifs, an L-box, rich with leucine residues and the B-box, containing a nuclear localization signal (NLS) required for transcriptional regulation.

After identifying StZFP2 as infestation- and wound-responsive [2], we used the potato genome [12, 13] to discover additional StZFP genes. Six additional StZFP genes map within 28,000 bases to each other on chromosome (chr) 11 in potato. In comparison, Arabidopsis and tomato only have three such orthologs. Tomato and potato both have 12 chromosomes and the three orthologs are also in the same region on chr 11 in tomato. It is estimated that potato and tomato formed separate species ~ 7.3 million years ago [14], so differences within the individual genomes would have evolved later. This work focuses on the five new StZFP2-like genes in potato, demonstrating that StZFP2-like transcripts are expressed, differentiating their expression in different tissues and upon infestation by the chewing insect pest *Manduca sexta*.

## Main text

### Methods

RNA was isolated using TRIzol per the manufacturer’s instructions [15]. After a final ethanol wash, Qiagen DNase and RNeasy columns were used to further purify the RNA. Integrity and concentration of RNA was determined with an Experion automated electrophoresis system for RNA (Bio-Rad).

Since infestation can perturb typical housekeeping genes [16] an exogenous control RNA was added to the cDNA synthesis step in lieu of an endogenous control. This approach has been used in several situations when no suitable reference gene can be found [17–19]. Luciferase control RNA (Promega) was added to the reverse transcription master mix. The cDNA synthesis reaction contained 2 µg template RNA and 2 ng luciferase RNA in 20 µl. This was diluted 1:20 with 10 µl used in the realtime reaction (50 ng RNA, and 50 pg luciferase RNA). Custom Taqman assay primer/probe sequences are listed (Additional file 1: Table S1). The qRT-PCR was performed using the 7500 Real Time PCR System (Life Technologies).

For the phylogenetic tree, proteins were aligned using CLUSTSALW and a tree was inferred using the Neighbor joining method [20]. The evolutionary distances were computed using the Poisson correction method [21] and are in the units of the number of amino acid substitutions per site 1000 bootstrap replicates were performed [22]. Evolutionary analyses were conducted in MEGA6 [23]. Alignment of ZFP proteins was performed using CLUSTAL OMEGA [24].

The plant material, *Solanum tuberosum* var. Kennebec (a common commercial variety) and insects were prepared as follows. At 2 weeks, tissue culture maintained rooted nodal plantlets were transferred to 3.5″ Arabipots (Lehle Seeds) containing Sunshine LC1 mix (Sun Gro Horticulture, Inc) for 19 days in a Conviron CMP6050 growth chamber with a 16:8 light/dark and 25 °C day/20 °C night temperature. The two youngest fully expanded leaves were excised and used for ‘control’ and ‘infested’ in feeding experiments. *Manduca sexta* larvae were reared to 4th instar on artificial diet [25] then transferred to Kennebec “feeder” plants in Arabipots for 18 h. Larvae were removed from plant material to empty petri dishes 2 h before assay.

Three-way ANOVA was conducted on the ΔC_t_ values [26] using SAS Proc MIXED [27] to accurately model among-genes and between-treatments. Pairwise means comparisons were conducted using the SLICE option of the LSMEANS statement to compare among times for each gene and treatment and to compare treatments for each gene at each time; using the Sidak method to adjust p values to protect against obtaining false positive comparisons. Letters indicating significant differences among means were generated using the pdmix800 SAS macro [28].

### Results and discussion

**Fig. 1:**
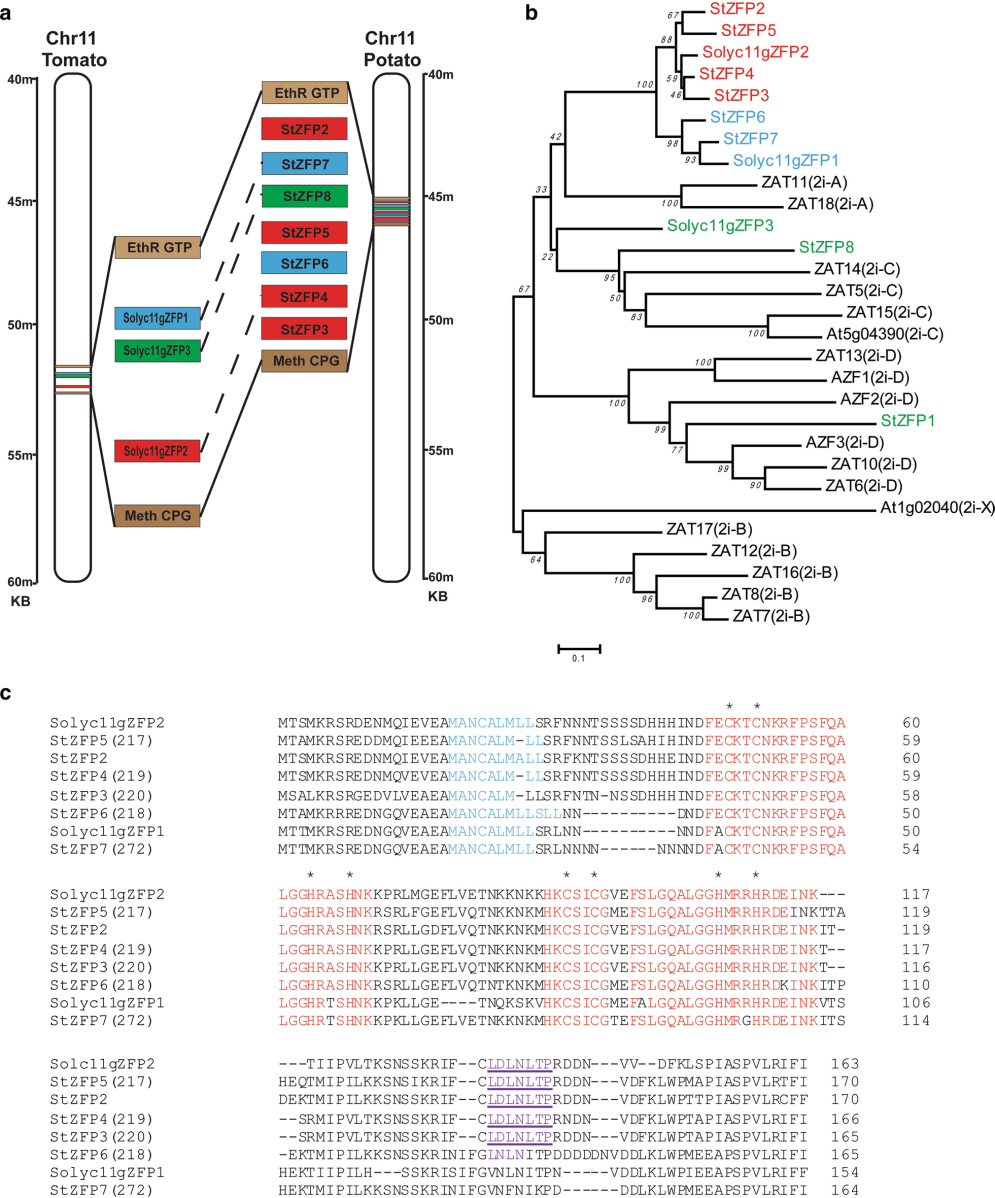
**a** StZFP2 and five homologs map to a 28 Kb region of chromosome 11. Another Q-type ZFP gene, StZFP8 (green) maps between the StZFP2-like cluster. The tomato orthologs in the homologous region of chromosome 11 are shown. StZFP6 and StZFP7 are orthologs of the tomato gene Solyc11gZFP1 (blue). StZFP2, StZFP5, StZFP4 and StZFP3 are orthologs of Solyc11gZFP2, which is currently unmapped in the tomato genome (red). Solyc11gZFP3 is a newly identified tomato ortholog of the gene StZFP8. Non-StZFP2-like genes EthR GTP and Meth CPG (brown), are present on either side of the StZFP2-like cluster in both tomato and potato. **b** Neighbor joining tree of Arabidopsis Zats, eight known StZFPs and three tomato ZFPs. Potato and tomato 2i-A proteins are divided into two clusters (red and blue). Five potato and two tomato StZFP2-like proteins cluster with 2i-A Zats. StZFP8 and Solyc11gZFP3 (green) clusters with 2i-C Zats and StZFP1 (green) groups with 2i-D Zats. **c** Alignment of StZFP2-like proteins. The L-box in blue, zinc finger domains in red with the invariant C2H2 amino acids marked with an asterisk. The Ear motif in purple. The top four StZFPs and Solyc11gZFP2 contain an EAR motif with both the canonical EAR motifs of LxLxL and DLNxxP described by Kagale and Rosenthal [24]

Using the StZFP2 protein sequence as a query, Blastp was used to search the Spud database [29]. The search identified the StZFP2, and six additional proteins. The names in the Spud DB database [30], and their map positions, are detailed in (Additional file 2: Table S2). StZFP2 gene maps to Chr11. The area around StZFP2 in the potato genome contains a cluster of genes that encode similar proteins (Fig. 1a). The ZFPs previously identified were StZFP1 and StZFP2 [2, 31]. While StZFP1 maps to chr 01 [31], the six potato StZFP genes form a 28 kb cluster on chr 11 (Fig. 1a). Two StZFP2-like orthologs are also found on chr 11 of the tomato genome and named Solyc11gZFP1 and Solyc11gZFP2. Their map position and database names are listed in (Additional file 3: Table S3). An additional StZFP gene (StZFP8) was found within the cluster on chr 11 and although this gene is not in the tomato database it was identified here, between the ZFP2-like genes and named Solyc11gZFP3 (Fig. 1a).

A phylogenetic tree (Fig. 1b) illustrates how the potato ZFPs compare to a family of similar proteins in Arabidopsis. The additional ZFPs in this work were named StZFP3 to StZFP8. The eight potato ZFPs, three tomato ZFPs residing on chr 11 and 18 Arabidopsis two fingered Q-type ZFPs (Zats) were aligned as described in “Methods”. Due to their conserved protein sequences, the 18 Arabidopsis ZFPs are considered C1-2i types, since they contain two zinc finger domains clustering into 2iA–D with an outlier X. Six of the 7 StZFPs, Solyc11gZFP1 and two clustered with the Arabidopsis Zat11 and Zat18 (2i-A), while StZFP8 grouped with tomato Solyc11gZFP3 and 2i-C Zats. StZFP1 clustered with the 2i-D Zats (Fig. 1b).

There were only two corresponding 2i-A genes in tomato (Fig. 1a) and Arabidopsis (Fig. 1b). StZFP6 and StZFP7 are orthologs of the tomato gene Solyc11gZFP1, while StZFP2, StZFP3, StZFP4 and StZFP5 are orthologs of a gene Solyc11gZFP2, which is not currently annotated on the tomato genome. The alignment of the protein sequences (Fig. 1c) confirms the phylogenetic tree, with the tomato protein Solyc11gZFP2 aligning with StZFP2-5 and the second tomato protein Solyc11gZFP1 aligning with StZFP6-7. Comparison of the protein sequence to tomato proteins showed that StZFP7 is closest to Solyc11gZFP1 while StZFP4 is closest to Solyc11gZFP2, suggesting that StZFP7 and StZFP4 existed prior to the split between the two species. The L-box, two zinc finger domains, and the EAR motif are also delineated (Fig. 1c). The EAR motif has been identified as an active repressor of genes that are bound by the ZFP zinc finger motifs [32] and the most common types are LxLxL and DLNxxP. The StZFP2-5 proteins all contain LDLNLP, which is a combination of these two classic EAR motifs.

Considering that there are StZFP2 and five StZFP2-like genes (2i-A) in potato, it is curious that there are only two in tomato and Arabidopsis (2i-A). The sequence read archive (SRA) [33] data for potato was queried and confirmed that all five transcripts were expressed. The most robust expression of these genes was in a sample of stolon tips rewatered after drought stress and, not surprisingly, the variety Igor after 24 h infestation by a chewing insect (Additional file 4: Figure S1). Thus, according to SRA data these genes are all expressed under stress conditions.

**Fig. 2:**
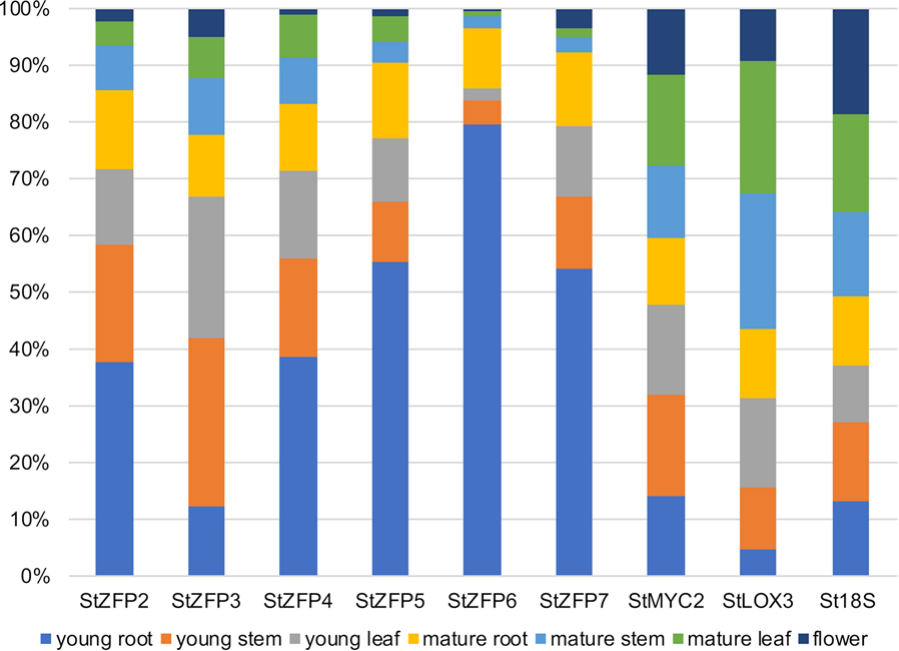
Expression of StZFP2-like genes in different tissues. Mean transcript level is 2^− dCT with dCT = CT of test gene-CT of exogenous control gene). Each gene is stacked to demonstrate the amount of expression in each tissue and is represented as a percentage of the total. Five Kennebec plants were dissected and pooled for each age and tissue type (root, stem, leaf, flower). Young plants were harvested on day 18 (approximately 2.5 weeks) at the four leaf stage. Mature plants were harvested on day 42 (approximately 6 weeks). The tissue was isolated under no known stress

qRT-PCR was used to determine if these genes were expressed in Kennebec. Primers identified the unique transcripts for StZFP2-7, with StMYC2 and StLOX3 marker genes for JA (Additional file 1: Table S1). StZFP2 and the five StZFP2-like genes were compared (Fig. 2). The least amount of StZFP expression was in flower and the greatest in young roots. StZFP3 was an exception with very low levels in all tissues except young stem and leaves. StZFP5 and 7 had similar profiles of tissue expression. StZFP6 had the highest expression in young root and the lowest in the leaf. In mature root the expression was similar for all StZFPs. Since basal levels in the root are higher for all but StZFP3, examination of root tissue may lead to a better understanding of the role the StZFP2-like genes play in potato.

**Fig. 3:**
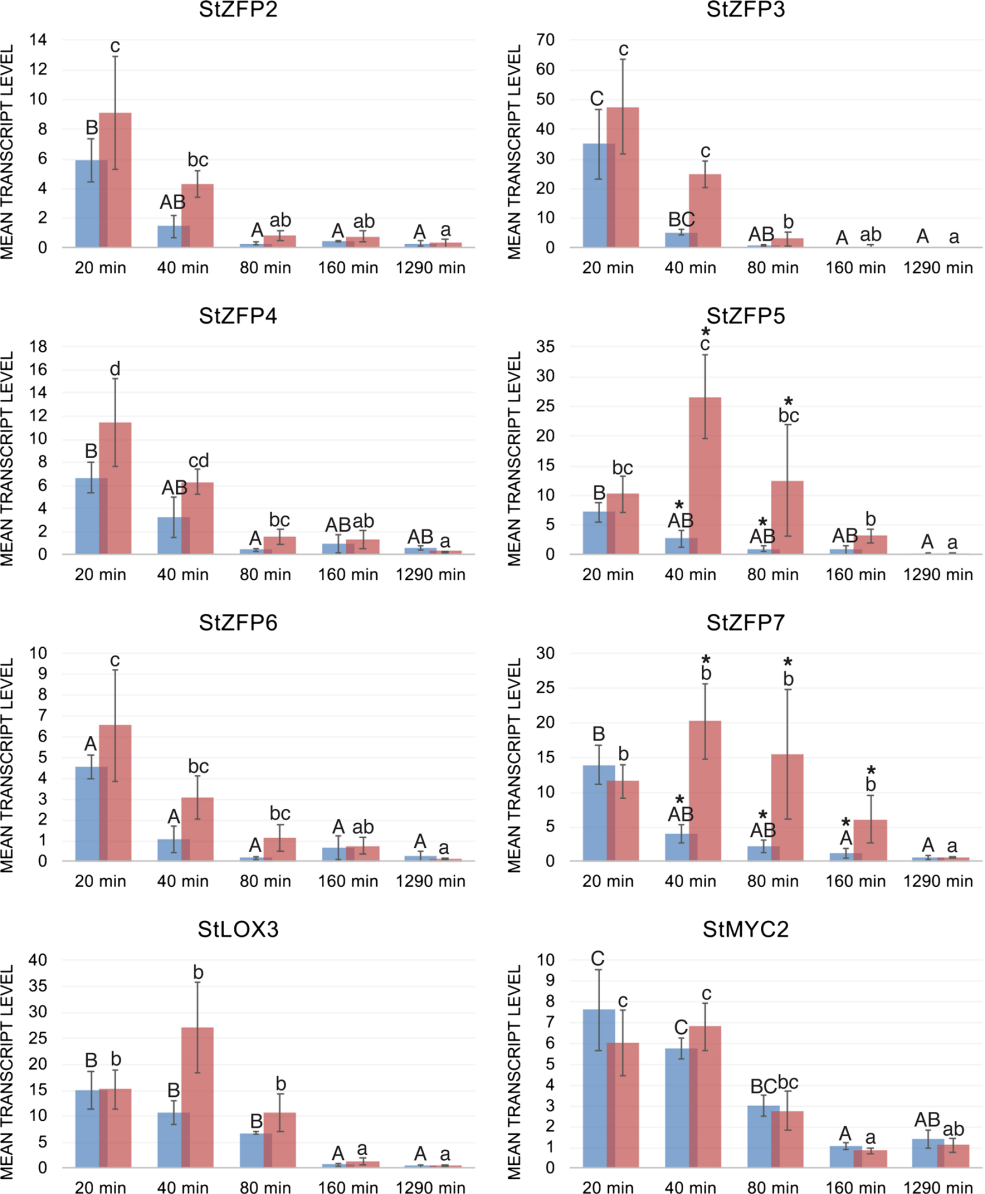
Expression of StZFP2-like genes in excised and leaves infested by *Manduca sexta*. Mean transcript level is, 2^− dCT with (dCT = CT of test gene-CT of exogenous control gene). Excised control leaves in (blue) and excised infested leaves in (red) are shown at each time point. Each value is the average of three biological replicates. Significant differences in control leaves over time are shown with capital letters. Significant differences in infested leaves over time are shown in lower case letters. Significant differences between control and infested leaves at the same time point is shown with an asterisk (*). Error bars represent standard deviation. Excised potato leaves from 19 day-old plantlets (cv, ‘Kennebec’) were infested with 3–4, 4th instar *Manduca sexta* larvae for 20 min. If any of the larva were not eating within 2–3 min, they were replaced with new larvae and at 20 min larvae were removed. Leaves were harvested (at 20, 40, 80, 160 and 1290 min) and immediately frozen in liquid nitrogen and stored at − 80 °C until RNA extraction. Leaf area consumed was calculated using Phenophyte [34], during a 20 min interval it ranged from 16 to 35%

qRT-PCR was used to examine whether the StZFP2-like genes were expressed upon excision and infestation by the chewing insect pest *M*. *sexta*. Excised leaves were subjected to infestation for 20 min, the larvae removed and the leaves harvested at the times indicated. Uninfested excised leaves were compared to infested at each time point (Fig. 3). The expression in detached leaves diminished earlier at 160 min while the infested leaves were not reduced until the final time point. StLOX3 and StMYC2 expression diminished by 160 min. StZFP2, StZFP3 and StZFP4 were significantly reduced in expression by 80 min in both detached and infested leaves. The expression of StZFP6 in infested leaves was significantly lower at 160 min, while replicates in the detached leaves were so variable that significant differences were not found. The expression of StZFP5 and StZFP7 were significantly higher in infested leaves versus detached at 40 and 80 min, with StZFP7 still higher in the infested leaves at 160 min. Therefore, while expression of StZP5 and StZFP7 were affected by detachment, infestation significantly increased their expression.

While StZFP2 and StZFP3 were similarly expressed by infestation, they had differences in tissue specificity. StZFP2 has ~ 50% of the young tissue expressed in the root and StZFP3 only ~ 18%. StZFP4 and StZFP6 are similarly expressed by infestation, but StZFP6 had twice as much expression in young root compared with StZFP4. StZFP5 and StZFP7 had similar tissue specific expression, but were unique in their response to infestation with StZFP5 falling at 160 min, and StZFP7 remaining significantly high at 160 min. Sequence similarity suggests that StZFP6 and StZFP7 were closest homologs, while StZFP2, StZFP3 and StZFP5 were derived from StZFP4. The expression of these genes does not appear to fit into these lineages. For example, the amount of expression in the roots for StZFP6 is high while StZFP7 is intermediate. The expression of StZFP6 by infestation decreases by 80 min while StZFP7 remains high until at least 160 min.

Initially, StZFP2 was identified as a gene induced by insect infestation [35]. Clearly, it is not the only ZFP in potato responsive to infestation. It is also not the most dramatically induced of the StZFP2-like genes. Why there are so many StZFP2-like genes in potato compared to Arabidopsis and tomato remains to be determined. It is predicted that the tomato and the potato genome contain approximately the same number of genes with 34,727 and 35,004 protein coding genes, respectively [36], so it is unlikely that the propagation of potatoes as tubers rather than through seed has simply allowed redundant genes to be retained in potato.

## Limitations

The StZFP2-like genes identified here are infestation induced and differ in their tissue specificity. Now that they have been identified, further research is needed to determine how the individual StZFPs affect the plants response to infestation. Perhaps over-expression of individual StZFP2-like genes may create different phenotypes with some more effective than others in improving resistance to insect pests with less negative effects on growth and development.

## Additional files

**Additional file 1: Table S1.** Taqman primers used in this work.

**Additional file 2: Table S2.** Q-type ZFPs in this work with potato genome reference name and location.

**Additional file 3: Table S3.** Tomato orthologs of potato genes from Fig. 1

**Additional file 4: Figure S1.** Alignments of the StZFP2-like transcripts detail the coverage of SRA data and confirm the expression of all unique genes.

## Abbreviations

JA: jasmonic acid
TF: transcription factors
ERF: ethylene-responsive element-binding factor
EAR: ERF-associated amphiphilic repression
NLS: nuclear localization signal
chr: chromosome
SRA: sequence read archive
ZF: zinc finger
