# Supplementary material for: The remarkable plethora of infestation-responsive Q-type C2H2 transcription factors in potato
Source: BMC Res Notes. 2018 Jun 19;11:398. doi: 10.1186/s13104-018-3503-6 (PMC6011193; doi:10.1186/s13104-018-3503-6)
Supplement: Supplementary file 1 — Additional file 1: Table S1. Taqman primers used in this work. [file 13104_2018_3503_MOESM1_ESM.docx]

**Table S1** Taqman primers used in this work

| **target** | **F primer** | **R primer** | **probe** |
| --- | --- | --- | --- |
| **luciferase** | AGAGCTGTTTTTACGATCCCTTCAG | GCGAAGAATGAAAATAGGGTTGGT | ACGCACTTTGAATTTT |
| **StZFP2** | AGTAGAGGCCATGGCTAATTGTG | CTGATGATGAAGAAGGTTTTTGAAACGA | CAACAGCGCCATTAAG |
| **StZFP3** | CTTCACTTCAAACTTCAAACTTTCACACA | CCTCTGCTTCTTTTTAAGGCACTCA | ATGAAGACACAAAGTTGTTTTT |
| **StZFP4** | TGTTCATTTTAGACATTTTGCATTCATTTTGT | TTTTTGTTTTTTAATTGAATCAAACTTTATTACGTTACTG | AAGAATGAACGAACTAATTAAG |
| **StZFP5** | CTCACATATTTTCAAACTTATATAATATACTAAAACATCT | CCTCTTCTTCAATTTGCATATCATCTTCTC | CATATTATTGATGAAACTAAAAATG |
| **StZFP6** | GCCTTTGGACTAAATGAATAGAAAGAATCC | GTCTTGCGAATATTTATTTAAGTAAAAAAGCTTATAATTTGTTAT | ACTGCATGTAGCTAATTAA |
| **StZFP7** | GGCTTGGAAAGATGGAAATCGTTTA | GCCTTAATGCTTTTGTCGCGTTTA | ACAATAACAACAACAATAACAATG |
| **StLOX3^*^** | CCAGTAAGCAAGCTTGATCCTGAAA | GACCAAGAATGTGCTCCTCCTT | ATGGCCCTCAAGAATC |
| **StMYC2^**^** | ATGTGAACTTGTTTACGGGTCAGT | CTGGAAGTAGCTGATCTTTTCTTGTTCT | CACTGCCCCAAATTG |

* StLOX3 is lipoxygenase 3. NCBI accession number X96406.1

** StMYC2 is a basic helix-loop-helix (bHLH) leucine zipper transcription factor. The primers were made to PGSC0003DMT400045204 from http://solanaceae.plantbiology.msu.edu/.
