# Supplementary material for: The remarkable plethora of infestation-responsive Q-type C2H2 transcription factors in potato
Source: BMC Res Notes. 2018 Jun 19;11:398. doi: 10.1186/s13104-018-3503-6 (PMC6011193; doi:10.1186/s13104-018-3503-6)
Supplement: Supplementary file 2 — Additional file 2: Table S2. Q-type ZFPs in this work with potato genome reference name and location. [file 13104_2018_3503_MOESM2_ESM.docx]

**Table S2** Q-type ZFPs in this work and potato genome reference name and location

| Gene Name | Genome gene name | Genome protein name | Chr location |
| --- | --- | --- | --- |
| StZFP2 | PGSC0003DMG400015557 | PGSC0003DMP400027271 | Chr11 45,238,065 to 45,238,765 |
| StZFP3 | PGSC0003DMG400015534 | PGSC0003DMP400027220 | Chr 11 45,265,448 to 45,266,065 |
| StZFP4 | PGSC0003DMG400015533 | PGSC0003DMP400027219 | Chr 11 45,260,834 to 45,261,488 |
| StZFP5 | PGSC0003DMG400015531 | GSC0003DMP400027217 | Chr 11 45,252,757 to 45,253,413 |
| StZFP6 | PGSC0003DMG400015532 | PGSC0003DMP400027218 | Chr 11 45,256,750 to 45,257,512 |
| StZFP7 | PGSC0003DMG400015558 | PGSC0003DMP400027272 | Chr 11 45,241,719 to 45,242,465 |
| StZFP8 | PGSC0003DMG400039582 | PGSC0003DMP400061686 | Chr 11 45,246,127 to 45,246,594 |
