# Supplementary material for: The remarkable plethora of infestation-responsive Q-type C2H2 transcription factors in potato
Source: BMC Res Notes. 2018 Jun 19;11:398. doi: 10.1186/s13104-018-3503-6 (PMC6011193; doi:10.1186/s13104-018-3503-6)
Supplement: Supplementary file 3 — Additional file 3: Table S3. Tomato orthologs of potato genes from Fig. 1 [file 13104_2018_3503_MOESM3_ESM.docx]

**Table S3** Tomato orthologs of potato genes from Fig. 1

| Tomato Genome Name | Tomato Gene Name Fig.1 | Gene location SL2.50 |
| --- | --- | --- |
| Solyc11g073050 | EthR GTP | ch11:56,164,984...56,168,738 |
| Solyc11g07360 | Solyc11gZFP1 | ch11:56,172,037…56,172,501 |
| Solyc11gZFP3 | Solyc11gZFP3 | ch11: 56,177,363…56,178,066 |
| Loc101261885 | Solyc11gZFP2 | ch11:56,181,014…56,181,713 |
| Solyc11g07080 | Meth CPG | ch11:56,185,857…56,189,288 |
