# Supplementary material for: The remarkable plethora of infestation-responsive Q-type C2H2 transcription factors in potato
Source: BMC Res Notes. 2018 Jun 19;11:398. doi: 10.1186/s13104-018-3503-6 (PMC6011193; doi:10.1186/s13104-018-3503-6)
Supplement: Supplementary file 4 — Additional file 4: Figure S1. Alignments of the StZFP2-like transcripts detail the coverage of SRA data and confirm the expression of all unique genes. [file 13104_2018_3503_MOESM4_ESM.docx]

**Figure S1** Coverage of SRA data for ZFP2-like potato transcripts

StZFP5 ATGACAGCCATGAAAAAAAGTAGAGAAGATGATATGCAAATTGAAGAAGAGGCTATGGCT 60

StZFP2 ATGACATCTATGAAAAGAAGCAGAGAAGACAATATGCAAATTGAAGTAGAGGCCATGGCT 60

StZFP3 ATGAGTGCCTTAAAAAGAAGCAGAGGAGAGGATGTGCTAGTGGAAGCAGAGGCCATGGCT 60

StZFP4 ATGACATCTATGAAAAGAAGCAGAGAAGACAATATGCAAGTTGAAGTAGAGGCCATGGCT 60

StZFP7 ---------ATGAAAAGAAGCAGAGAAGATAATGGACAAGTGGAAGCAGAAGCCATGGCT 51

StZFP6 ATGACAGCCATGAAAAGAAGAAGAGAAGATAATGGACAAGTGGAAGCAGAAGCTATGGCT 60

* **** *** **** *** ** * * * **** *** ** ******

StZFP5 AACTGCGCCTTGATG---CTTTTGTCTCGTTTCAACAACACTTCTTCATTATCAGCTCAT 117

StZFP2 AATTGTGCCTTAATGGCGCTGTTGTCTCGTTTCAAAAACACTTCTTCATCATCAGATCAT 120

StZFP3 AACTGCGCCTTAATG---CTTTTGTCTCGTTTCAACACCAACAA---CTCATCAGATCAT 114

StZFP4 AATTGCACCTTAATG---CTTTTGTCTCGTTTCAACAACACTTCTTCATCATCAGATCAT 117

StZFP7 AATTGCGCCTTAATG---CTTTTGTCGCGTTTAAACAATAAC-----AAC---------- 93

StZFP6 AATTGCGCCTTAATG---CTTTTGTCGCGTTTAAACAAG--------------------- 96

** ** **** *** ** ***** ***** ** *

StZFP5 ATTCACATTAATGATTTTGAATGCAAGACTTGTAATAAACGACTTCCGTCTTTTCAAGCC 177

StZFP2 CATGAAATTAATGATTTTGAATGCAAGACTTGTAATAAACGGTTTCCGTCTTTCcaagct 180

StZFP3 CATCATATTAATGATTTTGAATGCAAGACTTGCAATAAACGCTTCCCGTCTTTCCAAGCC 174

StZFP4 CATCACATTAATGATTTTGAATGCAAGACTTGCAATAAACGCTTCCCGTCTTTTCAAGCT 177

StZFP7 AACAATAACAATGATTTTGCATGCAAGACTTGTAATAAACGATTTCCATCTTTCCAAGCC 153

StZFP6 ------GATAATGATTTTGAATGTAAGACTTGTAATAAACAGTTTCCATCTTTTCAGGCT 150

********** *** ******** ******* * ** ***** ** **

StZFP5 TTAGGAGGTCATCGTGCAAGTCATAATAAACGATCAAGATTATTCGGAGAGTTTCTTGTT 237

StZFP2 ttaggcggtcacCGTGCAAGTCATAATAAACGGTCGAGATTACTCGGAGATTTTCTTGTT 240

StZFP3 TTAGGCGGTCATCGTGCAAGTCATAATAAAAAGCCAAGATTACTCGGAGAGTTTCTTGTT 234

StZFP4 TTAGGCGGTCATCGTGCAAGCCATAATAAAAAGCCAAGATTACTCGGAGAGTTTCTTGTT 237

StZFP7 CTTGGTGGACATCGTACAAGTCATAATAAAAAGCCAAAATTACTCGGAGAATTTCTTGTT 213

StZFP6 CTTGGTGGACACCGTGCAAGTCATAATAAACGGTCAAGATTACTAGGAGAGTTTCTTGTT 210

* ** ** ** *** **** ********* * * **** * ***** *********

StZFP5 CAAACCAATAAAAAGAATAAGATGCATAAATGCTCTATTTGTGGTATGGAGTTTTCTTTG 297

StZFP2 CAAACCAACAAAAAGAACAAGATGCATAAATGTTCTATTTGTGGTGTGGAGTTCTCTTTG 300

StZFP3 CAAACCAACAAAAAGAACAAGATGCATAAATGCTCTATTTGTGGTGTGGAGTTTTCTTTG 294

StZFP4 CAAACCAACAAAAAGAATAAGATGCATAAATGCTCTATTTGTGGTGTGGAGTTTTCTTTG 297

StZFP7 CAAACCAACAAAAAGAATAAGATGCATAAATGCTCTATATGTGGTACGGAGTTTTCATTG 273

StZFP6 CAAACCAACACAAAGAATAAGATGCATAAATGTTCTATTTGTGGTATGGAGTTTTCTTTG 270

******** * ****** ************** ***** ****** ****** ** ***

StZFP5 GGTCAAGCTTTAGGTGGACACATGAGGCGTCATCGTGATGAAATTAATAAAACTACAGCT 357

StZFP2 GGTcaagcattaggcggtcacATGAGGCGTCATCGTGATGAAATTAATAAAATTACAG-- 358

StZFP3 GGTCAAGCCTTAGGAGGGCACATGAGGCGTCATCGCGATGAAATTAATAAAACGT----- 349

StZFP4 GGTCAAGCCTTAGGAGGGCACATGAGGCGTCATCGCGATGAAATTAATAAAACGT----- 352

StZFP7 GGTCAAGCATTAGGCGGACATATGAGAGGTCACCGTGATGAAATTAATAAAATTACGTCT 333

StZFP6 GGTCAAGCACTAGGCGGACACATGAGGCGTCACCGTGATAAAATTAATAAAATTACAC-- 328

******** **** ** ** ***** **** ** *** ************

StZFP5 CATGAACAGACGATGATTCCGATTTTGAAGAAGTCAAATAGCATCAAGAGGATT------ 411

StZFP2 -ATGAAAAGACGATGATTCCGATTTTGAAGAAGTCAAATAGCAGCAAGAGGATA------ 411

StZFP3 -------CGAGGATGATACCAGTGTTGACGAAGTCGAATAGCAGCAAGAGGATT------ 396

StZFP4 -------CGAGGATGATACCAGTGTTGACGAAGTCGAATAGCAGCAAGAGGATA------ 399

StZFP7 CATGAAAAGACGATGATTCCAATTTTGAAGAAGTCGAATAGCAGCAAGAGGATTAATATT 393

StZFP6 -CTGAAAAGACTATGATTCCAATTTTGAAGAAGTCAAATAGCAGCAAGAGGATTAATATT 387

** ***** ** * **** ****** ******* *********

StZFP5 TTTTGTTTGGATTTAAACTTAACCCCTCGTGATGATAATGT---------TGATTTCAAG 462

StZFP2 TTTTGTttggatttaaacttaACCCCTCGTGATGATAATGT---------TGATTTCAAG 462

StZFP3 TTTTGTTTGGACTTAAATTTAACCCCTCGTGATGATAATGT---------TGATTTCAAG 447

StZFP4 TTTTGTCTGGACTTAAACTTAACCCCTCGTAATGATAATGT---------TGATTTCAAG 450

StZFP7 TTTGGGGTTAACTTTAACATAAAACCTGATGA------------------TGATTTGAAG 435

StZFP6 TTTGGGTTGAACTTAAACATAACACCTGATGATGATGATGACAATGTTGATGATTTGAAG 447

*** * * * ** ** *** *** * * ****** ***

StZFP5 TTATGGCCGATGGCACCAATTGCATCTCCTGTTTTGAGAACTTTTATTTAA- 513

StZFP2 TTATGGCCAACAACACCAATTGCATCTCCTGTTTTGCGATGCTTTTTTTAA- 513

StZFP3 TTATGGCCAACAACACCAATTGCATCTCCTGTTTTGAGAATTTTTATTTGA- 498

StZFP4 TTATGGCCGACCGCACCAATTGCATCTCCTGTTTTGAGAATCTTTATTTAAT 502

StZFP7 CTATGGCCGATGGAAGAGGCTCCATCGCCCGTCTTGCGAATATTTATTTAA- 486

StZFP6 TTATGGCCCATGGAAGAGGCTCCATCGCCCGTCTTGCGAATATTTATTTAA- 498

******* * * * **** ** ** *** ** *** *** *

**Figure S1.**  Alignment of the StZFP2-like transcripts Grey highlights represent 100% identical sequence to transcript data in either an SRA dataset or cloned transcript. Red nucleotides represent a different base compared to other ZFPs in the alignment. StFP5 transcript was identified in SRA dataset SRX502837, plant variety Igor 24hr infested with Colorado potato beetle; StZFP2 was cloned from wounded potato variety Kennebec and StZFP3,4,6 and 7 were found in SRA dataset SRX912192, Ningshu4 stolon tips rewatered after drought stress.
